# An analysis of the impact of digital technology adoption on the income of high quality farmers in production and operating

**DOI:** 10.1371/journal.pone.0309675

**Published:** 2024-09-03

**Authors:** Xiankai Lei, Dongmei Yang

**Affiliations:** 1 Business School, Xinyang Normal University, Xinyang, China; 2 Dabie Mountain Economic and Social Development Research Center, Xinyang Normal University, Xinyang, China; 3 School of Marxism, Xinyang Normal University, Xinyang, China; Zhongnan University of Economics and Law, CHINA

## Abstract

Digital technology is shaping our traditional agriculture in unprecedented ways. As a new engine to empower farmers and promote common prosperity in rural areas, the development of the digital economy can help revitalize rural industries, providing strong support for increasing industrial value-added income and farmers’ income levels. Based on 676 pooled cross-section data of high-quality farmers in China Guangdong Province from 2020–2021, the article empirically examines the impact of digital technology adoption on the production and business income of high-quality farmers and explores its mechanism of action, based on theoretical analysis and using the ERM model. It is found that the use of digital technology helps to increase the income of high quality farmers in production and business, but effects vary for different income levels and different types of high quality farmers. In addition, tests of the mechanism of action suggest that the introduction of digital technologies can mitigate the negative impact of market distance on the income of high quality farmers in production and business. After applying variable substitution, model replacement, and propensity score matching (PSM) for robustness checks, the research findings still hold true. Therefore, efforts should be made to speed up the upgrading of rural digital technology and other infrastructure, increase training for high quality farmers to improve their digital literacy through multiple channels, for the better marketing of agricultural products.

## Introduction

Promotion of farmers’ income is not only related to their immediate interests, but also to the steady progress of the rural revitalization strategy. Therefore, the promotion of farmers’ income, narrowing the gap between urban and rural incomes and achieving common prosperity has also been one of the key concerns of experts and scholars. There is a wealth of research results at home and abroad on the factors influencing farmers’ income, mainly in macro factors such as farmers’ digital literacy, agricultural subsidy policies, farmland property rights, rural infrastructure construction, agricultural extension and village governance capacity [1–4], as well as farmers’ personal capacity, non-farm transfer employment of rural labor and their gender micro factors [5–7]. The impact of market distance on farmers’ incomes is also a factor that cannot be ignored. China’s agricultural market faces the contradiction of "big market, small production", and farmers are still at a disadvantage in the traditional agricultural sales market, which, coupled with the increased transportation costs of agricultural products due to distance, inevitably inhibits the increase of farmers’ income [8–10]. In 2019, the 《Regulations on Rural Work in China》 first proposed the concept of "high-quality farmers", which is a group of farmers developed from new professional farmers, possessing the characteristics of "being educated, skilled in technology, good at management, and capable of business operation". Unlike ordinary farmers, high quality farmers are modern agricultural practitioners who are professionally engaged in agriculture, are technically proficient in business, and derive their income mainly from agriculture to a significant level [11].They mainly include three types: management and operation-oriented, professional production-oriented, and skill service-oriented. The characteristics of high-quality farmers are having cultural knowledge, understanding technology, being good at management, and skilled in operations. Since the issuance of the Central Document No.1 in 2012, which clearly stated the need to vigorously cultivate highly qualified farmers, the number has been rising rapidly. However, previous studies on high quality farmers have mainly focused on aspects such as definition and cultivation [12], leaving relatively few empirical analyses of their production and business incomes and their influencing factors. Only some scholars have noted that the status and individual experiences of high quality farmers can have an impact on their income [13, 14].

At present, high quality farmers gradually play a demonstration, radiation and driving role in China’s agricultural modernization and rural revitalization, and the effectiveness of their production and management also affects the implementation of the rural revitalization strategy [15]. According to a report on the development of high quality farmers released by the Ministry of Agriculture and Rural Affairs, the net income from agricultural operations of high quality farmers reached RMB 31,300,000 and RMB 33,000,000 in 2018 and 2019 respectively. Among them, more than 27% of high quality farmers in 2019 had a net income from their agricultural production operations higher than the per capita disposable income of urban residents in the same period. Relevant studies have confirmed that technological progress is a key factor in driving agricultural development and has a significant impact on agricultural production and business activities [16]. Online sales of rural agricultural products, for example, grew by 27% year-on-year to reach RMB 397.5 billion in 2019. Evidently, the “information dividend” brought by the Internet directly contributes to the increase of farmers’ income, but the impact mechanism varies [17–19],the main influencing mechanism is rural residents’ entrepreneurial or non-agricultural employment [20], and shows differential effects depending on the level of education [21]. Additionally, digital technology shows different effects on household labor resource allocation, farmland transfer out decisions, farm household entrepreneurial returns, and the degree of labor transfer [22–24]. It is fair to say that the digital economy is shaping our agricultural development and rural life, as well as farmers’ incomes, like never before.

Guangdong Province has always attached great importance to the cultivation of high-quality farmers. By the end of 2018, the number of high-quality farmers in Guangdong Province had reached 740,000. In 2019, Guangdong Province allocated 130 million yuan to support the cultivation of high-quality farmers. In 2020, the Department of Agriculture and Rural Affairs of Guangdong Province proposed to cultivate an additional 70,000 high-quality farmers in the next three years to promote the development of agriculture and rural areas. Thanks to good policy support and economic conditions, the number of high-quality farmers in Guangdong Province and the production and operation efficiency have experienced rapid development. So, In this paper, 676 questionnaire surveys of high quality farmers in China Guangdong Province were used to empirically investigate the impact of digital technology adoption on the production and business income of high quality farmers and the mechanism of its effect through ERM regression and moderating effect models, and further explore its heterogeneous impact on different income levels and different types of high quality farmers. The possible marginal contribution of this paper lies in the fact that (1) the study takes the adoption of digital technology by high quality farmers as the object of research to enrich the research results of high quality farmers;(2)The analysis framework integrates digital technology and market distance, comprehensively considering the impact of these two factors on the income of high-quality farmers’ production and operation. It also analyzes the market distance masking effect of digital technology on the income of high-quality farmers, providing a theoretical basis for further improving relevant systems to promote common prosperity among farmers; (3) The research methodology was chosen to take full account of the endogeneity issue and adopt the ERM estimation method, while the quantile regression method was used to make the findings of this paper more robust.

## Research hypothesis

### Digital technology and income from productive business of high quality farmers

Digital technology adoption can increase the income of high quality farmers in production and business. The specific role is reflected in the following aspects. Firstly, digital technology can increase the income of high quality farmers in production and management by bringing into play the effect of technological progress. The use of digital technology in agricultural production and management by high quality farmers provides a more rapid application of advanced production techniques to agricultural production and management, optimize factors of production allocation and facilitate growth in income from production and management. On the one hand, the development of digital technology has enabled new knowledge and technology in agricultural production and management to break through time and geographical restrictions, accelerating technological progress in agriculture and promoting changes in agricultural production methods. On the other hand, high quality farmers can acquire agricultural production and management knowledge at a relatively low cost, break the old knowledge system, update their knowledge of agricultural production in time, enrich their knowledge reserves and improve their production and management income.

Secondly, the use of digital technology can alleviate information asymmetry in the production of agricultural products, reduce transaction costs and increase the income of high quality farmers in their production operations. Production and marketing represent two of the most important aspects of the production and business activities of high quality farmers. In the traditional agricultural production model, the misalignment of information on the supply and demand of agricultural products can lead to a threat to the economic interests of high quality farmers. Digital technologies reduce the negative impact of information asymmetry through market information collection, screening and processing methods. The popularity and application of modern communication technologies, represented by the Internet, have greatly weakened or eliminated spatial and temporal barriers to accessing market information for high quality farmers, alleviating the problem of asymmetric production information and reducing the cost of searching for production information among high quality farmers [25]. At the same time, agricultural production is still largely a weak industry that " depend on Heaven for food", and early detection and action is an important option to avoid the risk of natural disasters in agriculture. The Internet, due to its speed of information transfer and the ease with which highly qualified farmers can access information, offers greater possibilities for highly qualified farmers to resolve possible losses caused by agricultural natural disasters, thus contributing to increased income for high quality farmers.

Thirdly, digital technology can help farmers achieve precise sales of agricultural products. By establishing e-commerce platforms or using mobile payment technologies, farmers can directly promote their agricultural products to the market and engage in direct communication and transactions with consumers. This not only reduces intermediate links and lowers sales costs but also improves sales efficiency and increases revenue. For example, the use of the internet breaks the time restrictions on agricultural product sales, allowing sales to take place around the clock. As a result, the timely information transmission of the internet and the "live streaming sales" greatly promote the sales of agricultural products and play an important role in resolving the difficulties in agricultural product sales and promoting farmers’ income growth. digital technology can also help farmers build agricultural product brands. By establishing online display platforms for agricultural products, farmers can showcase the unique features and advantages of their products, attracting more consumer attention and purchases. At the same time, utilizing social media and other channels for brand promotion can enhance the visibility and reputation of agricultural products, further increasing sales revenue. Additionally, digital technology can provide intelligent solutions for agricultural production management, helping farmers improve production efficiency and product quality. For example, using Internet of Things (IoT) technology and sensors to monitor soil moisture, temperature, and other information in farmland can assist farmers in scientifically regulating irrigation and fertilization, thereby increasing crop yield and quality. Furthermore, utilizing big data analysis and artificial intelligence technology can enable fine management of the production process of agricultural products, enhancing their safety and traceability, and increasing consumer trust and willingness to purchase. Additionally, the "live streaming sales" trend has also boosted the popularity of many "internet celebrity products," contributing to agricultural product brand building, increasing the value of agricultural products, and promoting the income growth of high-quality farmers. On this basis, the first hypothesis of this paper is formulated:

H1: The use of digital technology is conducive to an increase in the income of high quality farmers from productive operations.

### The market distance masking effect of the impact of digital technology adoption on the income of highly qualified farmers in production and management

On the one hand, the use of digital technology can mitigate the impact of market distance on the income of high quality farmers in their production operations. Agricultural producers are ultimately paid financially for their produce through the sale of their products on the market, and the state of the agricultural marketing chain is directly related to the economic returns of farmers. As times go by, the way agricultural products are traded between consumers and producers is also changing, from the traditional single sales channel to the current diversified sales channels, including the traditional producers going directly to agricultural markets, producers trading with the help of third parties (middlemen) and agricultural products traded through internet network platforms. Therefore, the improvement of markets such as the production and distribution areas of agricultural products and the establishment of stable marketing channels for agricultural products has been one of the key concerns of the top-level design.

According to the "cobweb theory", the lack of information on production by high quality farmers will increase the blindness of their production operations, making it difficult to match the supply of agricultural products with market demand, which may increase the uncertainty of transactions, leading to difficulties in selling agricultural products. This, coupled with the perishable nature of the produce, leads to higher transaction costs such as negotiation and lower income. Theoretically, the use of digital technology as a means of rapid information transfer can partially (not entirely) diminish the negative impact of geographical distance to markets on the income of highly qualified farmers in their production operations. Transaction cost theory provides a better perspective for the analysis of high quality farmers’ participation in agricultural market behavior in the context of the spread of digital technology. As the previous analysis shows, the distance from the agricultural sales market in the traditional trading model tends to result in a lack of market information for agricultural producers. Other things being equal, the greater the uncertainty in the trading environment and the probability of uncertainty in the behavior of the trading agents, the higher the transaction costs are likely to be.

**Fig 1:**
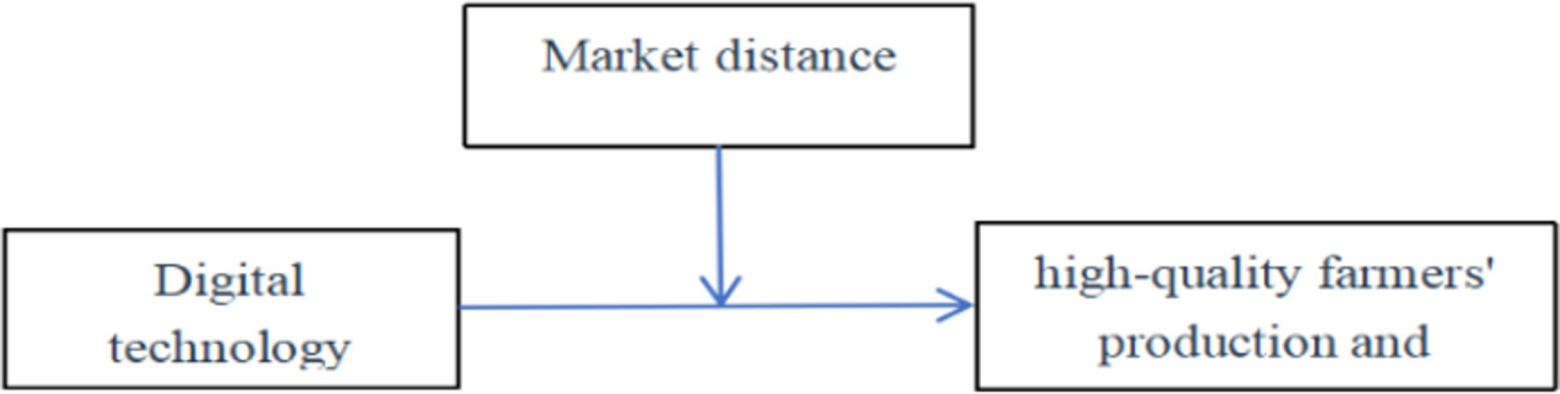
Theoretical analysis diagram.

In addition, the use of digital technology is breaking through the "distance gap" with a "digital dividend" and promoting the growth of productive income for high quality farmers. In the agricultural trade, the use of digital technology can break the shackles of geographical distance and eliminate physical barriers by means of diversification and digitisation, so as to break through the situation where it is difficult to sell agricultural products because of the distance from the agricultural sales market and, to a certain extent, alleviate the stagnation of agricultural products. It can also provide high quality farmers with accurate information about market demand, develop personalized marketing plans and increase the market share of their agricultural products. Trading agricultural products through online platforms is becoming one of the most important forms of trading today. This Internet sales model is mainly based on professional websites and other network sales, which can play the leverage effect, butterfly effect and eyeball effect to reduce the flow of agricultural products, broaden the sales channels of agricultural products, expand the sales range of agricultural products, reduce the negative impact of the geographical distance of the market on the sales of agricultural products, break through the "digital divide" and bring "information dividends" for the production and operation of high quality farmers (Fig 1). On this basis, the second research hypothesis of the text is proposed:

H2: Digital technology adoption can mitigate to some extent the negative impact of market distance on the income of new professional farmers.

## Data sources, variable selection and model setting

### Data sources

The data used in the paper comes from a questionnaire survey on high quality farmers conducted by the subject group in Guangdong Province in March and December 2020 (the surveys are for 2019 and 2020 respectively). Thanks to better policy support and economic conditions, the number of high quality farmers and production and business benefits in Guangdong Province have developed rapidly, reaching 740,000 by the end of 2018. According to the specific reality of each training site, the subject team took a random sample of high quality farmers who attended the training to ensure the quality of the data (each training session had participants from different municipalities). A total of 690 questionnaires were distributed and 683 questionnaires were collected. After removing the questionnaires with missing key values, 676 valid questionnaires were finally used in this study, with a questionnaire utilisation rate of 98.97%. The sample covered 20 prefecture-level cities (except Dongguan City) in Qingyuan, Guangdong Province. Based on the research focus of this paper, the content of the questionnaire survey mainly includes: (1) village level characteristics of high quality farmers, such as the geographical environment where the village is located, the construction of high standard farmland, the distance from the town government and the geographical distance from the market; (2) individual characteristics of high quality farmers, such as age, gender, education level, employment experience and training experience; (3) the production and operation status of high quality farmers, such as the scale of agricultural production and operation, type of agricultural production and operation, hired labour and agricultural insurance, etc.; (4) the use of digital technology, such as whether and to what extent the Internet is used in the sale of agricultural products, and whether the Internet is used to purchase agricultural production materials.

### Variables selection

Explained variable: income from productive business of high quality farmers. The explained variables, mainly measured by "income earned by high quality farmers from agricultural production activities in 2019 and 2020", include income from farming, livestock and aquaculture, etc. To lower the magnitude of the coefficient, the income variable was logarithmically treated. In addition, price levels vary from year to year because the paper uses two years of pooled cross-section data. In order to keep the variables consistent, income for 2020 has been treated according to 2019 income, after taking inflation into account.

Explanatory variable: digital technology adoption. Agricultural products are easy to wear out and difficult to store, and farmers have a higher rate of using the Internet to sell agricultural products after production than to buy agricultural production materials before production. Based on our research, more than 50% of high quality farmers choose to sell their produce using the Internet. Based on this, this paper focuses on the impact of the use of digital technology on the income of high quality farmers in the marketing of their agricultural products. Drawing on a study by Zhang and Zhu [26], the question "whether or not to use the Internet for product sales" was used as a proxy variable for the use of digital technology, with a value of 1 if the Internet was used, and 0 otherwise, based on the questionnaire set by the group.

Moderator variable: market distance. Farmers are ultimately paid financially through the sale of agricultural products in the market, but the agricultural market in China still runs into the contradiction of “big market, small production”, and farmers are always at a disadvantage in the sale of agricultural products with asymmetric information. Based on the study of Cai and Han [27], "proximity to the market" was chosen as a proxy variable for market distance.

Instrumental variable: Internet usage rate in the village. The impact of digital technology use on the production and business incomes of high quality farmers does not take into account the endogeneity of the problem, such as the possible reverse causality between digital technology use and the production incomes of high quality farmers. On the one hand, the demand for digital technology is generated by the higher income of high quality farmers; on the other hand, high quality farmers are more productive and more receptive to new things than the average smallholder farmer, and are more likely to adopt digital technology. Therefore, there may be an endogeneity problem. Referring to a related study by Zhao [22], "Internet usage in the village of the high quality farmer" was selected as an instrumental variable for whether the high quality farmer used digital technology. Endogenously, village Internet usage reflects the amount of Internet use by residents within a given area. From an exogenous perspective, it is difficult for the level of village internet usage to directly affect the income of high quality farmers from their production and business, and even if it does, the effect is based on whether or not high quality farmers’ households use the internet. Logically, "Internet usage in villages with high quality farmers" satisfies the instrumental variable.

Control variables. In addition to the impact that digital technology may have on the income of high quality farmers from production and management, the age and gender of high quality farmers and the number of people working in family farming may also have an impact on their income from production and management. Based on this, while considering the core explanatory variables, this paper introduces relevant control variables, mainly individual characteristics of high quality farmers, such as gender, age, education level, years of experience in agricultural production and whether they are cadres; household characteristics include the number of people working in household agriculture; differences in topographical characteristics, village infrastructure construction and distance from the township government also affect production and business income, so topographical characteristics of villages, the situation of water conservancy facilities and distance from the township government are also taken into account. The definitions of the variables and their descriptive statistics are shown in Table 1.

**Table 1:** Variable definitions, assignments and their descriptive statistics (N = 676).

Variable name	Variable meaning	Average	Standard deviation
Income from agricultural production and operations	Actual income (in logarithms)	4.36	1.63
Digital technology adoption	0 = No; 1 = Yes	0.53	0.50
Market distance	Geographical distance (km)	8.81	10.36
Gender	1 = female; 2 = male	1.76	0.43
Age	Actual age (years)	39.89	7.44
Education level	1 = Junior high school and below; 2 = Secondary or high school; 3 = College and above	2.12	0.68
Years in agriculture	Duration (years)	9.64	8.13
Village officials	0 = No; 1 = Yes	1.21	0.40
Number of people engaged in family farming	Number of people (persons)	2.25	1.16
Topographic feature	1 = plain; 2 = hilly; 3 = mountainous	2.41	0.77
Distance from town hall	Distance (km)	7.54	8.56
Water facilities to meet agricultural needs	1 = cannot be met, 2 = mostly can be met, 3 = can be met	1.69	0.64

### Sample cross analysis

Table 2 reports the results of the cross analysis of the sample. A cross analysis of 676 samples revealed that 359 samples of high quality farmers used digital technology in the process of selling their agricultural products, accounting for 53.11%, and their logarithmic value of income from agricultural production and operation was 4.61, higher than the full sample mean of 4.36; 317 sample of high quality farmers (46.89%) did not use digital technology in the marketing of their agricultural products, and the logarithm of their income from agricultural production operations was 4.07, indicating that the use of digital technology by high quality farmers in their agricultural production operations could increase their income. Gender differences in individual characteristics also differ in the production and business income of high quality farmers. 162 female samples of high quality farmers, representing 23.94%, were found to have a logarithmic value of 4.12 in their production and business income, while 514 male samples of high quality farmers, representing 76.06, had a higher production and business income of 4.43 than female high quality farmers. Among the topographical characteristics, there are differences in the economic effects due to the different topography. 117 of the sample of high quality farmers are in the plains (17.31%) and their logarithmic value of income from production and business is 4.90; 164 of the sample of high quality farmers are in the hills (24.26%) and their logarithmic value of income from production and business is 4.52; 395 of the sample of high quality farmers are in the plains, accounting for 58.43%, and the logarithm of their production and business income is 4.12.

**Table 2:** Sample cross analysis (N = 676).

Category		Production and operating income	Frequency	Percentage (%)
Digital technology adoption	Yes	4.61	359	53.11
	No	4.07	317	46.89
Gender	Female	4.12	162	23.94
	Male	4.43	514	76.06
	Plain	4.90	117	17.31
Topographic feature	Hilly	4.52	164	24.26
	Mountainous areas	4.12	395	58.43

### Model setup

The paper focuses on the mechanisms of the relationship between digital technology adoption and the productive income of high quality farmers. The adoption of digital technologies by high quality farmers is a self-selective process, and the decision to use may be influenced by unobservable factors, both internal and external to the individual, which in turn are related to the income of high quality farmers from their production and business, leading to possible selective bias in the model results. Also, the heterogeneity of resource endowments, etc. of high quality farmers at different income levels allows for the use of an extended regression model (ERM) to eliminate endogeneity and analyze the differences in the role of digital technology adoption on the production and business income of high quality farmers. The traditional instrumental variables approach is only applicable when the endogenous variables are continuous, whereas the ERM model is able to handle both continuous and discrete endogenous variables and is a more cutting-edge international approach to endogeneity problems. The income from productive business of high quality farmers is a continuous variable in this paper, thus Eregress is chosen for the regression and the model is constructed as follows:

$$LnY=\beta_{0}+\beta_{1}X_{1}+\beta_{2}X_{2}+\beta_{3}BDZ_{i}+\varepsilon_{\iota }$$ (1)

Where, *LnY* denotes the explanatory variable, which is the logarithm of income earned in the production business of high quality farmers, *X*_*1*_ is the market distance variable, *X*_*2*_ is the digital technology adoption variable and *BDZ* is the control variable such as individual characteristics.

## Empirical test results and discussion

### Baseline regression results

This paper presents a regression analysis of the impact of digital technology adoption on the productive income of highly qualified farmers using Stata 15 software. Before conducting the empirical test analysis, the first multiple cointegration test was conducted for digital technology adoption and control variables, etc. The results yielded a maximum VIF = 1.57, a minimum VIF = 1.03 and a mean VIF = 1.15, with the maximum VIF being significantly less than 10. It is clear that there are no serious problems of multicollinearity between the selected variables. The results of the ERM regression tests are reported in Table 3, where column (1) shows the regression results for the adoption of digital technology into the productive business income of high quality farmers, and column (2) shows the regression results for the inclusion of a series of control variables.

**Table 3:** ERM regressions of digital technology adoption on farm business income of high quality farmers (N = 676).

Variables	(1)	(2)
	Coefficient (standard error)	Coefficient (standard error)
Digital technology adoption	5.511^***^(1.444)	3.874^***^(1.156)
Market distance	-0.021^***^(0.006)	-0.022^***^(0.006)
Gender	‐‐‐	0.323^**^(0.140)
Age	‐‐‐	-0.016^*^(0.009)
Education level	‐‐‐	0.482^***^(0.088)
Years in agriculture	‐‐‐	0.003(0.008)
Village officials	‐‐‐	-0.332^**^(0.147)
Number of people engaged in family farming	‐‐‐	0.038(0.051)
Topographic feature	‐‐‐	-0.233^***^(0.072)
Distance from town hall	‐‐‐	0.008(0.007)
Water facilities to meet agricultural needs	‐‐‐	0.174^*^(0.096)
Cons	1.614(0.788)	2.044(0.902)

Note: *, ** and *** indicate significant at the 10%, 5% and 1% levels respectively.

Digital technology adoption helps to increase the income of high quality farmers in their productive operations. In both columns (1) and (2), the significance level of the effect of digital technology adoption on the productive income of high quality farmers is 1%, and the degree of impact decreases in both regression results with coefficients of 5.511 and 3.874 respectively. The empirical results are consistent with the expectations of the previous theoretical analysis, indicating that digital technology adoption can increase the productive income of high quality farmers. The possible reason for this is the masking effect on market distance through the technological progress effect or the information bonus of digital technology adoption on their income, which is also consistent with the results of numerous studies. Of course, whether this effect is differentiated by income level will be explored further below.

The market distance has a negative impact on the production and operation income of high-quality farmers. In the first column without controlling variables, the impact of market distance on the production and operation income of high-quality farmers is statistically significant at the 1% level. In the second column with controlling variables, the impact of market distance on the production and operation income of high-quality farmers remains significant, but the difference in coefficient size is small, at 0.021 and 0.022 respectively. This empirical result is consistent with the theoretical analysis in the previous text, indicating that market distance to some extent hinders the increase in production and operation income of high-quality farmers. The reason for this may be that the farther the distance to the sales market, the higher the transportation and transaction costs, which affects the sales radius and frequency of agricultural products, ultimately affecting the production and operation income of high-quality farmers. This suggests that in order to increase the income of high-quality farmers, it is necessary to address the issues brought about by market distance, reduce transportation and transaction costs, and expand the sales range and frequency of agricultural products

Control variables vary in their impact on the productive business income of highly qualified farmers. Control variables such as gender, age, education level and topographic characteristics of high quality farmers differ in the extent and direction of their impact on their productive business income. To further ensure the reliability of research findings. Specifically, gender, literacy and water infrastructure construction positively affect the income of high quality farmers from production and business at 5%, 1% and 10% levels of significance respectively; age negatively affects at 10% level; and topographical features negatively affect at 1% level, indicating that mountainous areas are to some extent not conducive to the increase of income of high quality farmers. Differences in control variables mean that the control for these factors may also boost the productive income of high quality farmers.

### Test for moderating effects

As theoretically analyzed earlier, the use of digital technology may attenuate the negative impact of market distance on the income of highly qualified farmers in their production operations. Based on this, a moderating effect model is adopted to test the pathways of digital technology adoption and market distance on the production and business income of high quality farmers, drawing on existing research findings. Table 4 reports the model results. In accordance with the traditional criteria for judging the moderating effect, the estimated coefficients and significance show that the coefficients of the effect of market distance differ between groups, verifying that the adoption of digital technology has a significant moderating role in the effect of market distance on the production and business income of high quality farmers, which also indicates that the use of digital technology mitigates the negative effect of market distance on the production and business income of high quality farmers.

**Table 4:** Results of the test for moderating effects (N = 676).

Variables	Production and operating income			
	Digital technology is not used		Digital technology is used	
	Coefficient	Standard error	Coefficient	Standard error
	OLS		OLS	
Market distance	-0.027^***^	0.009	-0.018^**^	0.009
Control variables	Controlled		Controlled	
cons	4.657	0.834	4.176	0.856
Sample size	317		359	

### Robustness tests

Substitution variable. In order to ensure the robustness of the findings, the article again estimates the impact of digital technology adoption on the production and business income of high quality farmers, using "whether or not to use platforms such as WeChat to sell products" as a proxy variable for digital technology adoption. The mean value of the variable "whether or not to use platforms such as WeChat to sell products" was 1.72. The findings show that both the direction of effect and the level of significance are more consistent with the results in Table 3, suggesting that the results of the impact of digital technology adoption on the income of high quality farmers in production and business are more robust (Table 5).

**Table 5:** ERM regression results for substitution variables (N = 676).

Variable	(1)	(2)
	Coefficient	Standard error
Whether to use platforms such as WeChat for product sales	5.160^***^	1.769
Control variables	Controlled	
cons	3.763	2.920

Replacement estimation method (OLS). Table 6 reports the results of robustness tests using replacement estimation methods. The choice of estimation method may also affect the research conclusions. To ensure the robustness of the results, this study uses the OLS model to estimate the impact of digital technology adoption on the income of high-quality farmers. The results show that both the direction and significance level of the effects are consistent with the results in Table 3, indicating that the impact of digital technology adoption on the income of high-quality farmers is robust.

**Table 6:** Impact of digital technology adoption on the income of high-quality farmers (replacement estimation method).

Variable	(1)	
	Coefficient	Standard Error
	OLS Model	
Digital Technology Adoption	4.157***	1.416
Control Variables	Controlled	
cons	2.341	
Sample Size	676	

Note: *, **, *** indicate significance at the 10%, 5%, and 1% levels, respectively.

PSM method. Table 7 reports the results of robustness tests using the PSM method. Due to data and variable limitations, and the fact that high-quality farmers’ adoption of digital technology in agricultural production and management activities does not meet the requirements of random sampling, but rather is the result of self-selection by new professional farmers, the analysis process still faces sample selection bias. Based on this, this study will use the propensity score matching (PSM) method to construct a counterfactual framework for correction, to verify whether the positive effect of digital technology adoption on the production and management income of high-quality farmers is consistent and stable. This study uses matching methods such as K-nearest neighbor matching and kernel matching to match the treatment group (high-quality farmers adopting digital technology) and the control group (high-quality farmers not adopting digital technology) based on propensity scores. The results show that the three matching results are similar, and all pass the significance test at the 1% level, with consistent effect direction and significance level. In addition, after matching using the three methods, the average treatment effect on the treated (ATT) is slightly reduced, with a small decrease in magnitude and consistent sign. Overall, the adoption of digital technology can promote the increase in production and management income of high-quality farmers. That is, after using the propensity score matching method to address endogeneity issues, the impact of digital technology adoption on the production and management income of high-quality farmers still has a significant promoting effect. This also indicates that the research results obtained in this study have not changed due to different matching methods, verifying the robustness of the empirical results on the impact of digital technology adoption on the production and management income of high-quality farmers.

**Table 7:** PSM estimation results for the impact of adopting digital technology on the productivity of new professional farmers.

Variable Name	Matching Method		Treatment Group	Control Group	ATT	Standard Error	T-value
Adopting Digital Technology	Kernel Matching	Before Matching	4.606	4.074	0.532	0.124	4.30
		After Matching	4.610	4.265	0.345	0.131	2.62
	K-nearest Neighbor Matching	Before Matching	4.606	4.074	0.532	0.124	4.30
		After Matching	4.610	4.204	0.406	0.155	2.60

### Further heterogeneous exploration

#### Income level considerations

The results of ERM regressions with high quality farmers’ farm business income as the explanatory variable and digital technology adoption, control variables, etc. are reported in Table 2 above. The baseline regression only examines the average effect of digital technology adoption on the productive income of high quality farmers, especially when extreme values of the productive income are found, making it difficult for the baseline regression to fully reflect the full impact of the independent variable on the dependent variable. To further analyze the impact of digital technology adoption on high quality farmers at different income levels, reference was made to a related study by Luo and Lei [15]. Based on the logarithmic values of the productive income of the surveyed high quality farmers, the productive income was selected to represent the low income level at the 0.25 quantile, the middle income level at the 0.50 quantile and the high income level at the 0.75 quantile, as a way to further investigate the heterogeneous impact of digital technology adoption on high quality farmers at different income levels. The regression estimation results are shown in Table 8.

**Table 8:** Impact of digital technology adoption on high quality farmers at different income levels (N = 676).

Variables	(1)		(2)		(3)	
	q = 0.25		q = 0.50		q = 0.75	
	Coefficient	Standard error	Coefficient	Standard error	Coefficient	Standard error
Internet use	0.232^**^	0.110	0.227^*^	0.128	0.612^***^	0.198
Control variables	Controlled		Controlled		Controlled	
cons	4.565	0.680	3.635	0.697	4.684	0.642

Digital technology adoption has a positive effect on production and business income for high quality farmers at low, medium and high income levels, but differences exist in the size of the effect, with digital technology adoption having a stronger effect on production and business income for high quality farmers at high income levels than for high quality farmers at medium and low income levels. Specifically, digital technology adoption passed the significance level test at the 0.25, 0.5 and 0.75 quartiles with coefficients of 0.232, 0.227 and 0.612 respectively, indicating that digital technology adoption is conducive to increasing the productive income of high quality farmers at low, medium and high income levels; In terms of significance and coefficient size, the increasing effect of digital technology adoption on the income of high quality farmers at high income levels is stronger than that of high quality farmers at middle and low income levels, which to some extent widens the income gap of high quality farmers. This may be due to the fact that the spread of digital technology has provided equal opportunities for high quality farmers to benefit from it, but this equal opportunity to benefit can have “dividend differences” depending on individual levels of awareness, income levels or digital technology adoption.

#### Considerations by type

Previously, the impact of digital technology adoption on the income of high quality farmers was analyzed. As different individuals, are there differences in the impact of digital technology adoption. Table 9 reports the impact of digital technology adoption on different types of high quality farmers. Based on the survey respondents and the definition of high quality farmers, there are four types of high quality farmers: large farmers, family farmers, farmers’ cooperative leaders and farmers’ entrepreneurs. In column (1), none of the effects of digital technology adoption on the farm production and business income of large farming households passed the significance test, indicating that their impact on the farm production and business income of large farming households was small. This may be due to: firstly, the lower use of digital technology by large farmers (39.39%), resulting in smaller economic benefits; and secondly, there is a larger scale of agricultural production operations by large farmers, which has a more pronounced scale effect and is less dependent on digital technology than traditional smallholders.

**Table 9:** Impact of digital technology adoption on the income of different types of high quality farmers in production and business.

Variables	(1)		(2)		(3)		(4)	
	Large breeder		Family farms		Farmers’ cooperative		Farmers’ agricultural enterprises	
	ERM		ERM		ERM		ERM	
	Coefficient	Standard error	Coefficient	Standard error	Coefficient	Standard error	Coefficient	Standard error
Digital technology adoption	5.003	3.420	0.907^**^	0.464	3.234^***^	1.158	4.624^**^	2.411
Control variables	Controlled		Controlled		Controlled		Controlled	
cons	3.518	1.904	3.924	0.747	4.654	1.446	0.039	2.191
Sample size	165		230		120		161	

In columns (2)-(4), there are significant impacts of digital technology adoption on the production and business income of high quality farmers such as family farms, but the extent of the impact varies. With regard to digital technology adoption, it has had the greatest impact on farmer cooperatives, followed by family farms and agricultural enterprises. Possible reasons for this are: firstly, the adoption of digital technology has to some extent increased the human and social capital of family farmers, reduced the transaction costs and business risks of agricultural products, and contributed to the increase in income from agricultural production and operations; secondly, based on the characteristics of China’s family farms, which, relative to developed countries such as the United States, have the characteristics of small scale production and operation and flexible operation, the adoption of digital technology can effectively shorten the production cycle of agricultural products, improve the delivery capacity of agricultural products, and make small batch and boutique agricultural products reach consumers more quickly and directly through the e-commerce platform; thirdly, the adoption of digital technology can promote innovative production and business practices in agricultural enterprises, break through the limitations of traditional agricultural operations and guarantee the quality of agricultural products while reducing production costs. In addition, digital technology adoption makes it possible to trace the origin and quality certification of agricultural products, maintain agribusiness brands and bring about a premium in the brand value of agricultural products.

## Conclusion and implications

Based on theoretical analysis, the paper uses ERM and moderating effect models to empirically test the impact of digital technology adoption on the production and business income of high quality farmers based on 676 questionnaire survey data in Guangdong Province from 2020–2021, and to explore whether digital technology adoption can change the impact of market distance on production and business income. The findings show that, in general, digital technology adoption can increase the income of high quality farmers’ production and business, but this impact varies across income levels and types of subjects; the greater the distance to market, the greater the resultant increase in transport costs and the more detrimental to the income of high quality farmers’ production and business; and the impact of control variables on the income of high quality farmers’ production and business varies. After a further robustness test by adopting substitution variables, the conclusion that digital technology adoption still has an impact on the productive income of highly qualified farmers is robust; the moderating effect goes some way to suggesting that internet use can mitigate the negative impact of market distance on the productive income of high quality farmers.

Clearly, to further increase the income of high quality farmers in production and management, first of all, high quality farmers should take into account the importance of digital technology in agricultural production and management and actively adopt and use digital technology to narrow the "digital divide" brought about by the spread of digital; secondly, the government should continuously increase the capital investment in the construction of rural digital technology infrastructure and logistics facilities, accelerate the upgrading of rural digital technology and other infrastructure, reduce the cost of digital technology use by high quality farmers, and increase the probability and efficiency of digital technology use by high quality farmers in agricultural production and operation; thirdly, training on the use of digital technology for high quality farmers should be increased in a multi-channel and targeted manner, so as to "digitally empower" and "digitally strengthen" high quality farmers, raise their awareness and level of digital technology use, enhance their ability to use digital technology in various aspects of agricultural production, marketing, processing and transportation, reduce market information search and transaction costs, enable them to earn higher incomes from agricultural markets, and alleviate the widening income gap; finally, efforts should be made to improve the sales channels for agricultural products, further bring into play the positive effects of digital technology, establish diversified sales channels for agricultural products, innovate the sales model for agricultural products, expand the brand influence of premium agricultural products and reduce the negative impact of geographical distance from the market on agricultural production and operation.

## Supporting information

S1 Data (XLSX)
